# Behavioral patterns in robotic collaborative assembly: comparing neurotypical and Autism Spectrum Disorder participants

**DOI:** 10.3389/fpsyg.2023.1245857

**Published:** 2023-10-26

**Authors:** Marta Mondellini, Pooja Prajod, Matteo Lavit Nicora, Mattia Chiappini, Ettore Micheletti, Fabio Alexander Storm, Rocco Vertechy, Elisabeth André, Matteo Malosio

**Affiliations:** ^1^Institute of Intelligent Industrial Technologies and Systems for Advanced Manufacturing, National Research Council of Italy, Lecco, Italy; ^2^Department of Psychology, Catholic University of the Sacred Heart, Milan, Italy; ^3^Department of Human-Centered Artificial Intelligence, University of Augsburg, Augsburg, Germany; ^4^Department of Industrial Engineering, University of Bologna, Bologna, Italy; ^5^Scientific Institute, Istituto di Ricovero e Cura a Carattere Scientifico E. Medea, Lecco, Italy

**Keywords:** human-robot collaboration, Autism Spectrum Disorder, Industry 4.0, behavior analysis, joint activity, wellbeing

## Abstract

**Introduction:**

In Industry 4.0, collaborative tasks often involve operators working with collaborative robots (cobots) in shared workspaces. Many aspects of the operator's well-being within this environment still need in-depth research. Moreover, these aspects are expected to differ between neurotypical (NT) and Autism Spectrum Disorder (ASD) operators.

**Methods:**

This study examines behavioral patterns in 16 participants (eight neurotypical, eight with high-functioning ASD) during an assembly task in an industry-like lab-based robotic collaborative cell, enabling the detection of potential risks to their well-being during industrial human-robot collaboration. Each participant worked on the task for five consecutive days, 3.5 h per day. During these sessions, six video clips of 10 min each were recorded for each participant. The videos were used to extract quantitative behavioral data using the NOVA annotation tool and analyzed qualitatively using an *ad-hoc* observational grid. Also, during the work sessions, the researchers took unstructured notes of the observed behaviors that were analyzed qualitatively.

**Results:**

The two groups differ mainly regarding behavior (e.g., prioritizing the robot partner, gaze patterns, facial expressions, multi-tasking, and personal space), adaptation to the task over time, and the resulting overall performance.

**Discussion:**

This result confirms that NT and ASD participants in a collaborative shared workspace have different needs and that the working experience should be tailored depending on the end-user's characteristics. The findings of this study represent a starting point for further efforts to promote well-being in the workplace. To the best of our knowledge, this is the first work comparing NT and ASD participants in a collaborative industrial scenario.

## 1. Introduction

The constantly increasing deployment of collaborative robots (cobots) in industries has led to a growing body of literature focused on achieving safe and effective human-robot interaction. Human-Robot Collaboration and Human-Robot Interaction are concepts highly related to the understanding of human cognitive behavior (Hormaza et al., 2019), and many issues still need to be tackled when the wellbeing of an operator inside a collaborative cell is taken into account (Nicora et al., 2021).

Stress, repetition, fatigue, and work environment are the cause of 48% of the variance of human error in manufacturing scenarios (Yeow et al., 2014), thus it is crucial to observe and evaluate which characteristics related to the cobot and which traits and conditions of the user may influence these factors. Moreover, to the best of our knowledge, no analysis has been published up to now involving adults characterized by the Autism Spectrum Disorder (ASD) working in a collaborative assembly cell, even though many aspects of the said collaboration may be beneficial for this group of individuals. The fixed and predictable routine with precise task assignment (Goris et al., 2020) that characterizes the collaborative work with a cobot represents a great inclusion opportunity (Hendricks, 2010). Considering such a scenario, it is important to remember that the behavioral patterns elicited by neurotypical operators (NT) are expected to be different from the ones of operators characterized by ASD. Depending on the autism features of each specific operator, each situation that may occur during a workday could lead to different and unexpected reactions which need to be considered at the time of task assignment.

Further analysis is necessary to ensure that the wellbeing of each worker is respected. As highlighted by emerging research, this is crucial due to the potential benefits that working with technology could bring for workers with ASD in terms of inclusion (Hendricks, 2010; Kagermann and Nonaka, 2019). Moreover, the emphasis on flexibility and customization in Industry 4.0 (Michaelis et al., 2020) underscores the importance of considering individual needs. Furthermore, the constantly growing paradigm of Industry 5.0 is paving the way for user-centered and user-oriented design of workplaces with the goal of transitioning to a more sustainable and human-centric industry. For these reasons, this study aims to draw a qualitative and quantitative comparison between the behavioral patterns elicited by NT participants and participants characterized by ASD during a generic collaborative assembly scenario. To the best of our knowledge, this is the first work comparing NT and ASD individuals in a collaborative industrial scenario, making it a promising study in the field, with positive benefits in terms of inclusiveness and mental health.

This work aims to observe the behavioral manifestations of the participants and measure their performance, to try to understand their experience during an assembly task in an industrial scenario. These observations and suggestions will allow us to better understand the overall experience and in particular tiredness and stress, in order to be able to anticipate this state of overload in the future and adapt the experience to the user accordingly. Furthermore, the interest of the present study is to observe any differences between neurotypical and ASD participants in the interaction experience. After presenting an overview of the literature on the topic in Section 2, the proposed collaborative assembly scenario is described in Section 3.1. The study protocol followed for this analysis is reported in Sections 3.2, 3.3, 3.4 and 3.5. Then, the main behavioral patterns observed for NT participants are presented in Section 4.1 while those of the participants characterized by ASD are described in Section 4.2. Finally, the results of qualitative and quantitative comparison between the two experimental groups are reported in Section 4.3 before a final discussion and some conclusive remarks in Section 5.

## 2. Background

We are recently witnessing a transition from an automation phase to a phase of effective collaboration with robots (Weiss et al., 2021), but examples of human-robot interaction with a high level of collaboration are, at the moment, still quite rare in real industrial environments (Michaelis et al., 2020). The term “collaborative robots” encompasses multiple levels of collaboration, ranging from coexistence to joint object manipulation (Aaltonen et al., 2018). With the increasing complexity of the interaction, a more sophisticated level of understanding of social signals, human needs, and the characteristics of the individual is required since the cobot must understand and adapt to human actions (Inkulu et al., 2021). Moving in this direction, recent research studies aim to evaluate and explain human behavior in interaction with collaborative robots.

For example, Toichoa Eyam et al. (2021) used some physiological parameters measured by electroencephalographic signals (EEG) to evaluate the human emotional state (stress, involvement, and concentration), and consequently adjusted some parameters of the cobot with which they are assembling a small wooden box. The goal was to keep these subjective variables in a desirable range to create a human-robot interaction characterized by a sense of security and trust. Michalos et al. (2018) implemented a robotic system for the assembly of an object in which the robot takes care of moving the heaviest materials. They emphasized usability and intuitiveness. The user was equipped with a smartwatch and Augmented Reality glasses to exchange information with the cobot, leading to a reduction in the execution time of the task and an ergonomic benefit for the user. Similarly, in El Zaatari et al. (2019), the goal was to reduce human tension and boredom. Thus, cobots moved and held large pieces, completed repetitive and precise tasks, and assembled parts that were difficult for humans to access.

Furthermore, studies have explored the utilization of users' gaze behaviors to enhance human-robot collaborations, with a primary focus on improving throughput. For example, Huang and Mutlu (2016) and Shi et al. (2021) used the user's gaze as a means of communicating choice. Their setup involved the robot picking the pieces selected by the user through their gaze. Huang and Mutlu (2016) showed that collaboration performance improves when the robot can anticipate the user's choice based on their gaze behavior. In Mehlmann et al. (2014), a robot capable of tracking the user's referential gaze was shown to speed up a collaborative sorting task, reduce the number of attempts, and require fewer clarifications to resolve ambiguity. Some works (Saran et al., 2018; Prajod et al., 2023) also demonstrated that gaze can be used to infer the attention of the user during human-robot collaboration.

All the studies presented up to now consider neurotypical adults, while a lack of knowledge can be found when considering human-robot interaction scenarios involving adults with ASD. This is particularly true for industrial applications even if the existing literature suggests that such scenarios could represent a beneficial inclusion opportunity for this group of individuals. For instance, the American Psychiatric Association (1994) provides an interesting discussion stating that repetitive and stereotyped behaviors are representative features of the autistic disorder. Social skills deficits (Weiss and Harris, 2001), a preference for predictability (Goris et al., 2020), difficulties in transitioning (Sterling-Turner and Jordan, 2007) and the need for concrete external feedback on personal performance (Larson et al., 2011) are other relevant aspects that characterize the autism condition. Starting from these considerations, it is possible that the working routine required for industrial automated tasks matches some of the needs listed before, specifically when considering the high-functioning part of the spectrum of the autism disorder (Gillberg, 1998).

As mentioned, however, industrial applications are not well researched in this sense and most of the researchers tend to use robots to help children with ASD in social integration, rehabilitation, and skills development, which seems to improve the cognitive and social skills of these users (Ghiglino et al., 2021; Saleh et al., 2021; Silva et al., 2021; Chevalier et al., 2022). For example, Panceri et al. (2021) and Baraka et al. (2022) employed social robots to enhance the therapy outcomes and improve the children's engagement during the sessions. Similarly, Lytridis et al. (2022) demonstrated that the LEDs on a social robot can be effective in engaging children during therapy sessions.

Some of the recent studies investigated whether the individual differences of children with ASD influence their behavior during human-robot interaction. Schadenberg et al. (2021) investigated the children's visual attention (where they look) and behavioral engagement (carrying out the activity) as a response to variances in robot behavior. They found that predictability in the robot's behavior positively influences visual attention. Whereas, behavioral engagement was influenced by the severity of autism features and expressive language ability. Lee and Nagae (2021) evaluated the distance that the children with ASD maintain while interacting with a social robot. Irrespective of the severity of ASD, the children were within a personal distance (typically between family or friends) from the robot.

The present work does not aim to build a theory on the characterization of ASD and neurotypical participants during the proposed experience, i.e., the assembly of a gearbox; rather, we aim to observe the behavioral manifestations in the two groups of participants, in a context that has so far been investigated very little. However, we expect differences to emerge between the two groups, starting from the evidence in the literature of some differences between ASD and neurotypicals in different activities. For example, it's known that subjects with ASD are more likely and frequently to demonstrate stereotypical movements with their hands (Gonçalves et al., 2012), or that they have less adaptive capacity and problems of planning inflexibility (Rajendran et al., 2011). Our study will help us to better understand if and what differences will emerge between the two groups, to better outline the needs of different users. In this sense, it is important to first understand the differences between the needs of NT and ASD participants in these kinds of scenarios in order to be able to provide a positive tailored experience. Given the innovative nature of our study, we have chosen an exploratory and observational approach, as further detailed in the Section 3.

## 3. Materials and methods

### 3.1. Collaborative assembly task

**Figure 1:**
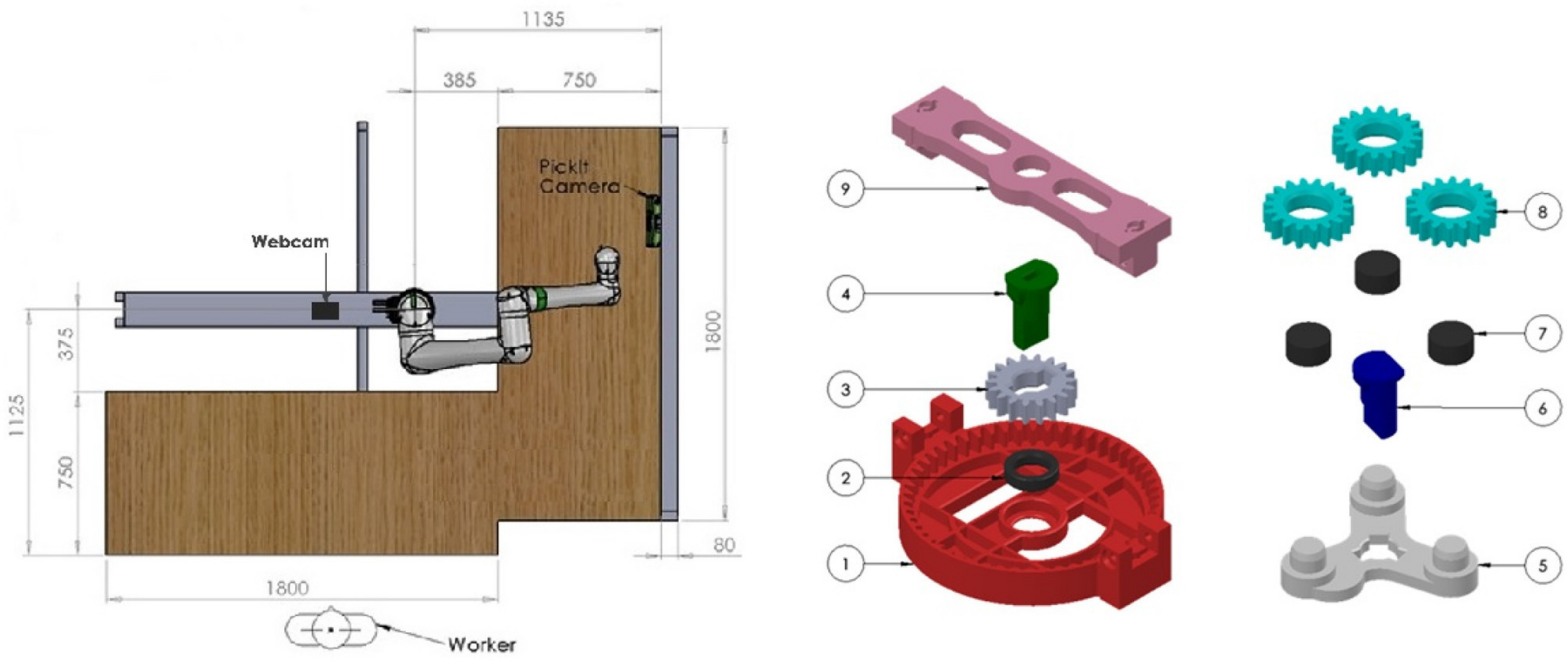
**(Left)** A schematic overview of the experimental workcell is depicted. **(Right)** The components that make up the complete assembly.

**Figure 2:**
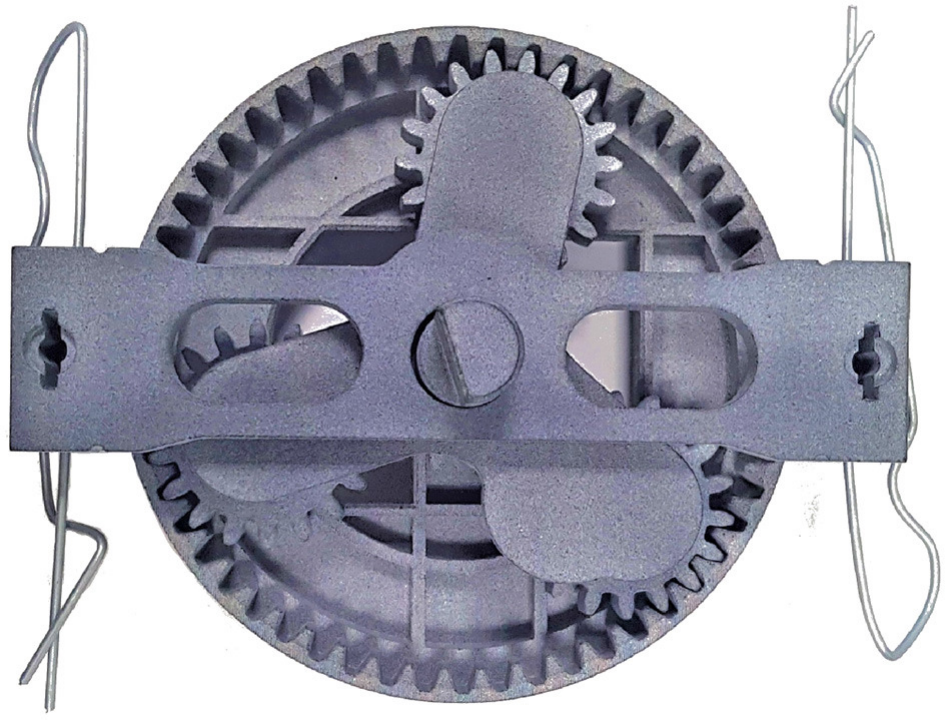
A picture of the finished product. The gearbox is made up of all the components depicted on the right side of Figure 1 plus two clips that keep the assembly together.

A generic collaborative assembly scenario is set up in a lab-based environment to obtain a deeper understanding of industrial operators' habits and experiences. The product to be assembled is a 3D printed planetary gearbox (Redaelli et al., 2021). With reference to the right side of Figure 1, half of the components (1–4) are put together by the cobot, while the human participant assembles the remaining parts (5–9). If needed, the participant can use an *ad-hoc* designed support structure. Once done with its part, the cobot moves toward the common area and stops in front of the user while keeping the sub-assembly at a convenient angle to facilitate the meshing of the gears. The two sub-assemblies are then joined collaboratively to obtain the finished product depicted in Figure 2. As the meshing is complete, the participant presses a pedal to trigger the robot to release the gearbox and start a new production cycle. Notice that the user also must make sure that the cobot always has enough spare parts on its table to be able to keep assembling by replenishing the buffers that are running low using the components provided in nearby boxes.

For this experiment, a Fanuc CRX10iA/L collaborative robot is mounted on a structure specifically built to guarantee a fixed relative position with respect to two tables arranged in an L-shaped formation, as represented in the left side of Figure 1. The table on the right is equipped with all the components required for the sub-assembly assigned to the cobot, together with a Pickit3D camera, used for the detection of parts. The table on the left is where the participant performs most of the activities and also where the collaborative session of the task takes place. The whole system is driven by a control architecture integrating ROS (Quigley et al., 2009), for controlling both the detection camera and the cobot, and Visual Scene Maker (Gebhard et al., 2012), used for the definition of the assembly steps and the synchronization of the different software modules.

### 3.2. Participants

This study is performed with 16 participants, of which eight were NT (five females and three males, 18–30 years old) and eight were diagnosed with high-functioning ASD (one female and seven males, 21–50 years old), meaning the absence of intellectual disability (IQ>70). We can observe an unbalance in the sex distribution toward males for the ASD group, as expected from literature (Loomes et al., 2017). It is also important to note that none of the participants had prior experience working with an industrial cobot.

Participants were asked to work on the task for 3.5 h a day, for five consecutive days, in order to capture and observe modifications in their performance and behavior during the overall experience (from Monday to Friday). Given the extensive duration of the experimental sessions, they were recruited considering their availability to autonomously reach the lab where the experiment takes place (by train or by car) or to spend the entire week in a nearby facility. Moreover, to facilitate the participation of ASD individuals, they were all briefed about the people they may interact with, the task to be carried out, and the daily procedures of the lab (e.g., security checks, lunch breaks, etc.) before the start of the experimental week.

### 3.3. Session recordings

A camera is set up in front of the participants to record them during the experimental activities. For this purpose, a Logitech C920 Pro HD webcam is used, and videos are recorded in 1,280 × 720 format at 25 fps. Since the experimental activities require the participants to move around in the workspace, the camera positioning is designed to keep the user in frame with a frontal view for as long as possible. As shown in Figure 1, the webcam is placed to the left of the cobot, on the available support structure, and around 1.5 m from the participant.

Three sessions of ~ 10 min each are video-recorded during the first workday (beginning, middle, and end of the workday). Likewise, three additional videos were acquired during the last workday of the experiment. Thus, 1 h of videos for each participant can be analyzed, for a total of 16 h of videos, to outline a qualitative and quantitative analysis of the behavioral patterns elicited by both NT and ASD participants.

### 3.4. Ethical approval

The study is conducted according to the guidelines of the Declaration of Helsinki and approved by the Ethics Committee of Istituto di Ricovero e Cura a Carattere Scientifico Eugenio Medea (protocol code N. 19/20CE of 20 April 2020).

### 3.5. Measures

Given the lack of knowledge highlighted in Section 2 regarding behavioral patterns elicited during industrial collaborative applications, especially for operators characterized by ASD, the authors decided to opt for a mixed-method approach for the analysis. Four different tools were used to collect robust measures that could be representative of both predictable and unforeseen behaviors. Some of the chosen tools allow for the precise observation of predefined aspects of the collaboration but are not suited for the analysis of long sessions (e.g., video-based annotations). Other tools, instead, have been selected for their good fit with long and unpredictable scenarios (e.g., live note-taking). Moreover, the different chosen measures allow for both a qualitative analysis of the observed behaviors for each experimental group and a quantitative comparison between the two mentioned groups. Note that the available quantitative measures have only been used in terms of comparison since they are specific to the chosen scenario and therefore have limited value in terms of absolute measures. Below, a detailed description of the four selected tools is reported.

#### 3.5.1. The observational grid

As mentioned before, one goal of the present study is to observe and try to understand the behaviors of the participants during the interaction with the robot, in particular relating to wellbeing and performance. To detect some of those predictable aspects, an observational grid is built. The grid is a tool that helps the observer remember and measure the goals s/he has set for himself. It consists of a table to record the observable events relating to the constructs of interest (Roller and Lavrakas, 2015). Given the nature of this approach, it is best suited for the precise observation of relatively short experimental sessions, and it was therefore applied for the analysis of the collected videos. The choice fell on this tool as it would have allowed the observer to record the observable events with respect to some categories of our interest (which will be described below) and the key areas consistent with the specific task proposed (Roller and Lavrakas, 2015). To build the grid, we decided originally to note the observed manifestations related to four attitudes: (1) “manifestations of tiredness,” (2) “gestures with the hands” (3) “assembly methods,” and (4) “loading pieces on the cobot table.” With “**manifestations of tiredness**” we mean those body movements or facial expressions that convey to the observer that the participant is tired. We chose this category as the ultimate aim of the project is to create an experience that tires the user with ASD as little as possible, and we were therefore interested in understanding whether tiredness is manifested in different ways and quantities in the two groups of participants. With “**gestures with the hands**,” we note all the hand movements that are frequent but not useful for the task (for example, touching the nose). It was our interest to check whether, even in this scenario, ASD users showed different hand movements in terms of modality and quantity compared to ASD participants, as happens in other contexts (Gonçalves et al., 2012). The “**assembly methods**” class encompasses how the participant assembled the planetary gearbox, for example, using one or both hands, building several pieces at the same time, etc. The last variable, “**loading pieces on the cobot table**,” refers to when the participant chose to supply the cobot table with new pieces, intended as the moment of the process and not as a chronological time; examples of this variable are “when the cobot stops,” “at any time,” “when the participant finishes assembling a gearbox.” These two categories were interesting for us, knowing that subjects with ASD have rigidities in changing their behavior while carrying out the same task; we, therefore, wanted to observe whether this difficulty was present in the two activities of assembling and positioning the gearbox components. After having examined the videos for the first time, other categories deemed important to explain the behavior of the participants are added: (5) “**other manifestations**,” which include other behaviors that cannot be categorized as due to tiredness, but which contribute to describing the moment e.g., fanning the shirt for the heat; (6) “**regard for the cobot**,” which includes reactions related to the behavior of the cobot (e.g., staring at it, talking to it) and also no reactions (e.g., ignoring that the cobot has been waiting for the joint action to happen). This category, initially overlooked, was proposed after the first visualizations of the videos as correspondence was noted with what had already been noted in the literature, namely a special interest in using computer-based programs on the part of ASD individuals. Moore et al. (2005); (7) “**talk to someone**,” in case the participant talks to someone in the room. On top of these variables a “notes” column is used by the researcher to add any additional observations made while looking at the collected videos.

Although this grid does not claim to categorize the participants' behaviors, it has proved to be useful for observing some patterns that we consider relevant during the experience and that can guide us in our observation. An example of the final version of the grid with data related to one of the participants is reported in Table 1.

**Table 1:** An example of a filled-in grid used to note the behavior of one of the participants.

**ID**	**4014006**
Day	Day 1 Video 2
Manifestation of tiredness	Participant looks at the clock (1.35; 10.25)
Gestures with the hands	Scratch the nose (4.10) Scrub hands (6.38)
Assembly methods	–
Loading pieces on the cobot table	–
Other manifestations	Tight lips (8.38) wet mouth with tongue (0.36; 0.58; 6.31; 7.20; 9.23)
REGARD FOR The cobot	Cobot arrives, user prefers to finish assembling all their half gearboxes
Talk to operator	Yes
Notes	Rubs hands after completing action, as satisfaction

#### 3.5.2. Unstructured notes

**Figure 3:**
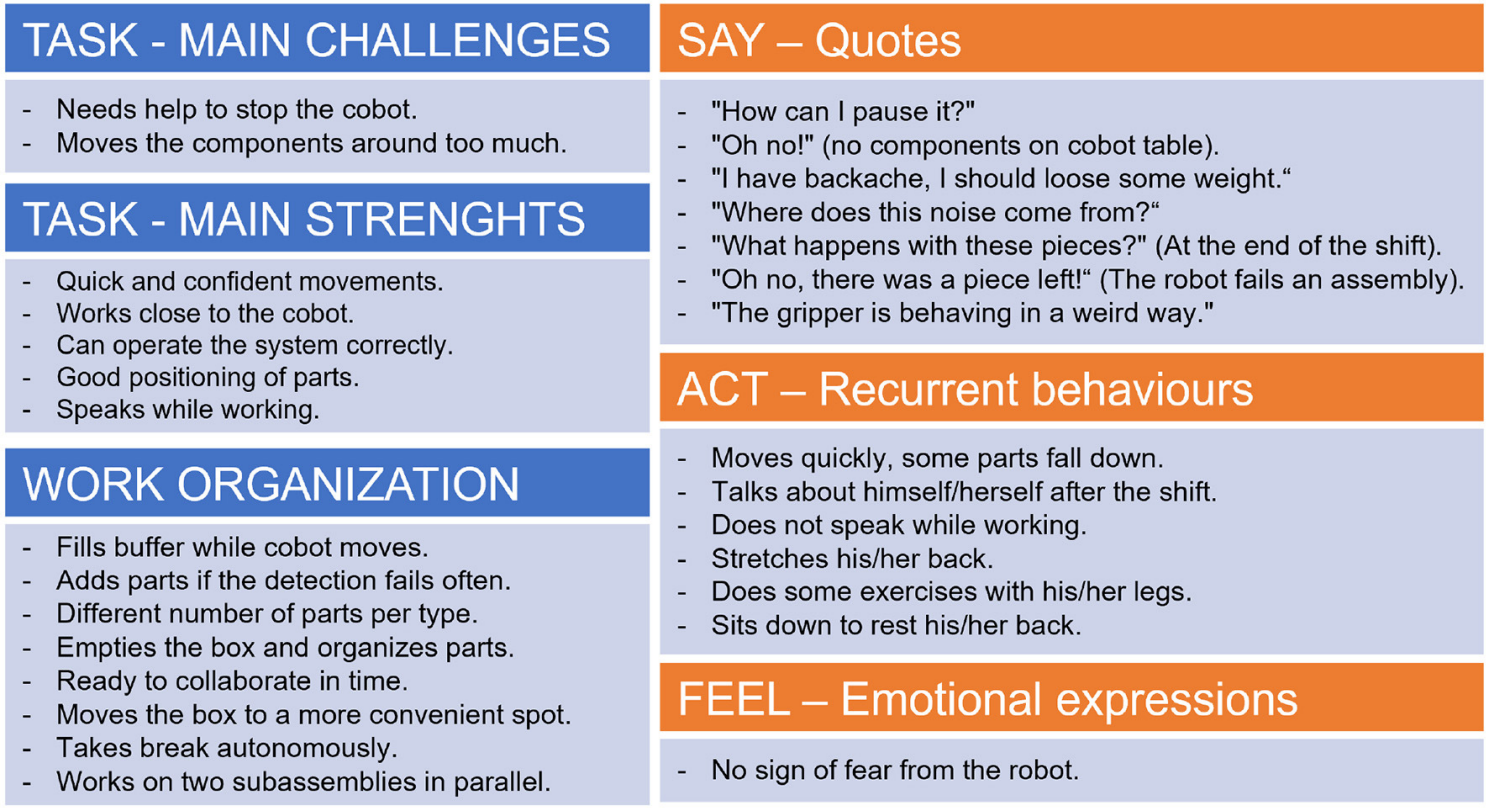
Example of a Persona compiled for one of the ASD participants.

Still today, the diagnosis of autism is based on behavioral markers. Each individual with ASD is likely to have a unique pattern of behavior (in some cases even stereotypical) which tends to be stable over time, still showing common signs that (from low to high functioning) lead to the formulation of a common diagnosis. Considering these premises on the importance of behavior, for the ASD group, we decided to collect additional data in the form of unstructured note-taking to make sure that the loss of specific behavioral occurrences is minimized. Therefore, during the 1-week experiment, two researchers performed a field observation, taking unstructured notes about the human-cobot interaction happening in the lab setting. Specifically, out of 5 days, three work shifts (lasting 3.5 h each) were observed: usually on Mondays, Wednesdays, and Fridays. The logic behind this choice was to picture the beginning, the middle, and the end of the week to see if any substantial change occurred over time. For the entire shift, the researchers (sitting at a desk from a distance and observing the participants non-intrusively), typed down on a computer what was going on while seeking to avoid influencing events occurrence. Unstructured notes were collected without any a priori grid, thus offering the possibility to catch any additional information that was not previously planned and that might happen outside the recording sessions. According to the deductive thematic analysis, the researcher, driven by specific interests, explores the dataset to code the information according to a preexisting theoretical framework or preconceptions (Nowell et al., 2017; Kampira and Meyer, 2021). Operationally, the researcher collected all the text files, grouping each by participant and specifying whether the notes were taken during the first, second, or third day. Then, by adapting the empathy map (a tool used in UX design to succinctly characterize each user Nielsen Norman Group, 2018), a qualitative profile of each ASD participant in the research was drawn up. Informative cards, named “Personas,” (see the example provided in Figure 3) were compiled, summarizing the profile of each ASD participant in five categories: “Task” (divided into “main challenges” and “main strengths”), “Work organization,” “Say—quotes,” “Act—Recurrent behaviors” and “Feel— Emotional expressions (if any).” By the “**Task**” category, we mean the main challenges and strengths that occurred between the cobot and the operator during each phase of the assembly task (e.g., s/he is able to manage the cobot stops, s/he is aware of the pedals usage, s/he is concentrated on the task, etc.). By “**Work organization**” we intend for example the strategies used by the operator to fill the tables with the corresponding pieces or the ability to manage some operations simultaneously. The last three categories “**Say—quotes**,” “**Act—Recurrent behaviors**” and “**Feel—Emotional expressions (if any)**” refer to what participants verbalized during the assembly task, the recurrent actions (not strictly related to the assembly task, e.g., checking the phone, crossing the arms, snapping the fingers) and eventually any kind of emotional expression (e.g., smiling, singing, etc.). It is important to note that the two researchers responsible for this tool were different from those who filled the observational grids described above. Also, it is important to reiterate that since the information was collected without the observer systematically searching for a specific behavior (as was done through the Observational Grid), it was not possible to perform a frequency quantification but only a qualitative description of the emerged behaviors. In this paper, we addressed the research need to outline the behavioral peculiarities of ASD participants; hence, unstructured notes were collected 3.5 h a day for the duration of three work shifts. The unstructured notes were collected only on the ASD group, as the researchers aimed to describe as much as possible the novelty of neurodiverse participants interacting with collaborative robots: being ASD a condition manifesting in behavioral patterns, the researchers wanted to picture any peculiarity or unexpected work-method during the experiment. This kind of information could not be collected through the predefined grid, as the duration of the videos was limited (compared to the 3.5 h per three days observations in the lab setting) and the observable events were defined a priori. Therefore, the decision to use PERSONAS only for ASD participants supported our aim to use an exploratory and qualitative approach to view the data more extensively, rather than to make a comparison between groups.

#### 3.5.3. The NOVA annotation tool

NOVA (Heimerl et al., 2019), also known as the NOn Verbal Annotator, is a tool designed for annotating and analyzing behaviors in social interactions. NOVA has a graphical interface, which provides a user-friendly way to annotate multimodal data. This data can come from various sources and sensors such as video, audio, and bio-signals. Also in this case, this tool is particularly suited for the annotation of relatively short experimental sessions and it was leveraged for the quantitative analysis of the videos recorded by the frontal camera. One of the annotation methods offered by NOVA is frame-wise labeling. This means that researchers can mark specific moments in the data to identify and categorize different behaviors. In addition, the interface is customizable and can handle data corresponding to multiple individuals or entities in separate tracks. This allows for the analysis of interactions between different entities, in our case, the interactions between a participant and the cobot. In addition to its annotation and visualization capabilities, NOVA annotations can be exported to popular formats, such as Excel. In our case, the annotations are saved in the following format: Start time, End time, and Label.

**Figure 4:**
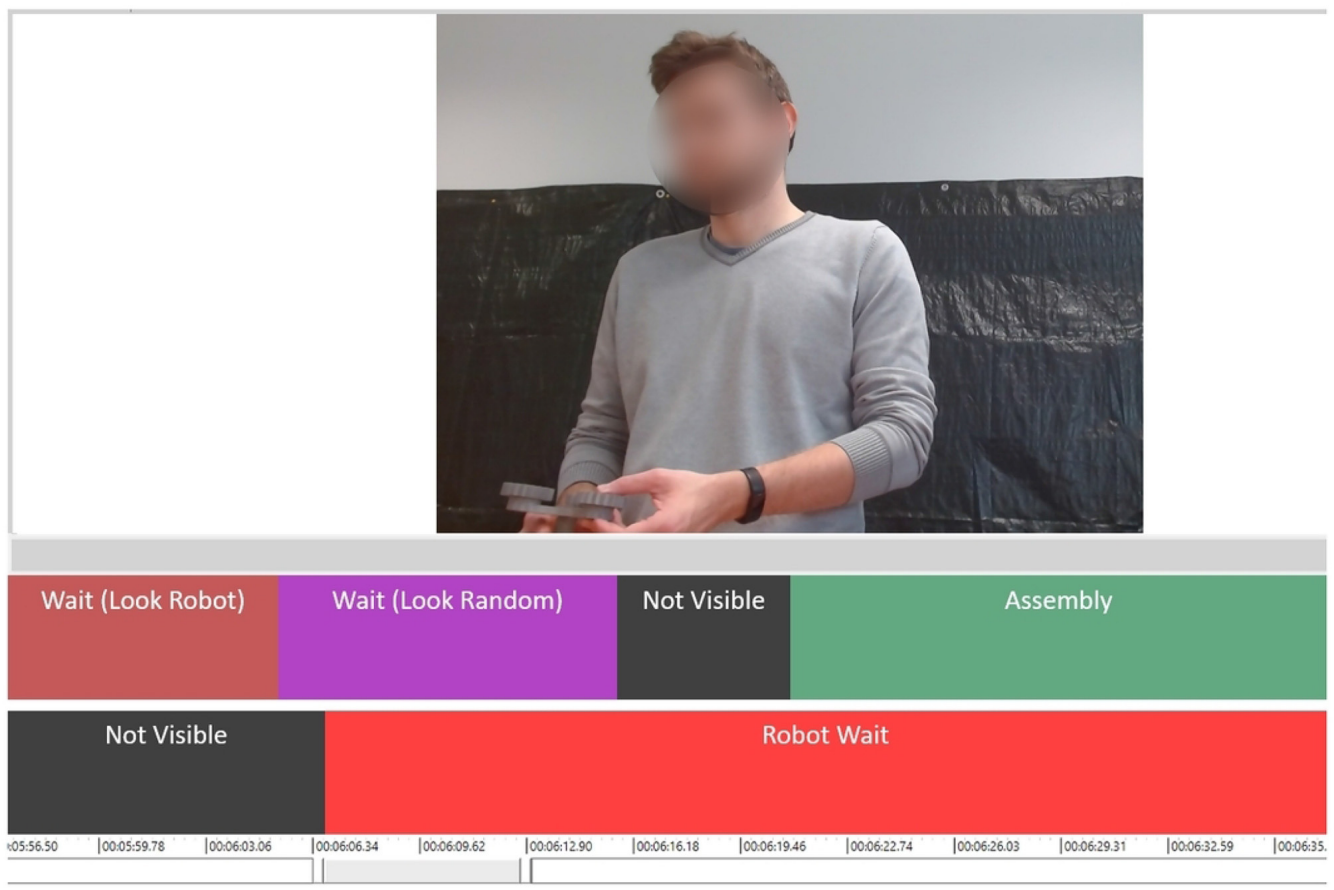
An illustration of session annotation for a participant. The image shows a video frame of the participant waiting for the robot. The top track has the labels for the participant and the one below has labels for the robot.

To quantify the duration of specific actions and compare the differences between NT and ASD participants, we utilized the NOVA tool for video annotation. Our labeling process involved two tracks of labels: one for the participant and one for the robot, as depicted in Figure 4. The task primarily consisted of three activities from the participant's side—gathering components, assembling them into a sub-assembly, and the final joint assembly involving both the participant and the robot. Consequently, these three actions were included in our label list as “**Gathering**,” “**Assembling**” and “**Final Joining**,” respectively. Additionally, we incorporated labels for waiting, both from the participant's perspective and the robot's perspective. During waiting periods, we observed a common pattern of participants looking at the robot. Hence, we distinguished between waiting while looking at the robot and waiting while engaging in other activities, such as looking in random directions, talking to someone, or other distractions. The two types of a participant's waiting behaviors are labeled as “**Wait (Look Robot)**” and “**Wait (Look Random)**.” Unlike the other actions, the robot's waiting (“**Robot wait**”) is an action of the robot rather than the participant (see Figure 4). However, the duration of the robot's wait depends on how the participants did their tasks and their decision on when to do the final joining. Notably, in the videos, a portion of the robotic arm was visible when it brought its sub-assembly, allowing us to label the moments of the robot waiting for the participant and the occurrence of the final joining. Due to the specific actions required in the task, participants would occasionally move to areas that were not captured by the camera. These instances were labeled as “**Not visible**” to indicate when the participant's movements extended beyond the purview of the camera. Given this, we had a total of seven labels, comprising task-related actions, waiting actions, and participant visibility. These labels provided the annotation scheme to capture and analyze the participant behaviors exhibited during the task. These labels and the corresponding durations will be used to compare the differences between the two groups of participants (Neurotypical and ASD). We will employ independent samples *t*-tests or similar tests (Mann–Whitney test, Welch test, etc.) to determine if these differences are significant. However, it is important to acknowledge that the small sample size may impose inherent limitations on both statistical power and the ability to detect small or medium effects.

#### 3.5.4. Full-week performance analysis

One piece of information that is missing from the data that is possible to collect using the tools presented up to now is the quantitative performance achieved by each participant during the whole experiment. Therefore, for every day of the experimental week, the researchers noted on an Excel sheet the start and end time of the session, any occurring stop of the activity, and the total number of assembled gearboxes per day. An overall performance analysis is then carried out in terms of the number of completed assemblies per hour. To do so, only the actual up-time (active working time) is considered. In fact, within the 3.5 h per day during which the participants worked on the task, some downtime occurred both in terms of breaks (requested by the participants) and in terms of unexpected stops (e.g., robot failures that required a restart of the system). By computing the ratio between the daily number of completed assemblies and the corresponding daily up-time, a performance index was computed for all the members of the two groups over the whole experimental week. The trend of downtimes will also be considered to rule out any bias that may affect the mentioned performance measure. Moreover, to compare the performance index trends of the two groups, a first check over the normality of the data distribution will be done using the Shapiro-Wilk test. If the normality assumption is verified, we intend to perform an ANCOVA analysis on the dataset to check the time*group influence effect over the performance. Again, it is important to keep in mind that the statistical power of the performed analyses may be impacted by the small available sample size.

## 4. Results

This Section reports the results obtained through the analysis of the behavior of NT and ASD participants together with a final comparison of the main observations extracted for each group.

### 4.1. Neurotypical participants

#### 4.1.1. Results from the observational grid

As mentioned in Section 3.5, the Observational Grid was employed to track the occurrences of the constructs of interest and how many participants (*N*, out of the eight individuals of the group) exhibit this behavior.

The results are summarized in Table 2 and are explained in more detail in the Supplementary material, paragraph 1. However, it is possible to briefly mention some suggestions that have emerged that are not captured by the mere table.

**Table 2:** Summary of the observed behaviors related to the NT group.

	**First day**	**Last day**
Manifestation of tiredness	- Lean hands or arms on table while waiting cobot (*N* = 5) - Movements of hands (*N* = 3) - Hands on hips (*N* = 2) - Sit (*N* = 1) - Time monitoring (*N* = 3) - Stretch (*N* = 2)	- Lean hands or arms on table while waiting cobot (*N* = 8) - Sit (*N* = 1) - Time monitoring (*N* = 4) - Stretch (*N* = 1) - Yawn (*N* = 2) - Snort (*N* = 1)
Gestures with the hands	- Rub fingertips (*N* = 1) - Rub face (*N* = 4) - Rub hands (*N* = 2) - Touch hair (*N* = 3) - Pull up the sleeves of the sweatshirt and adjust clothes (*N* = 1) - Touch glasses/watch (*N* = 1)	- Touch hair (*N* = 3) - Rub face (*N* = 6) - Touch glasses (*N* = 3) - Tap the watch (*N* = 1)
Assembly methods	- Start assembling the gearbox as the participant take out the useful parts from the box (*N* = 3) - Empty the whole box before the assemblation (*N* = 1) - strategy changed - Sequential assembly (*N* = 3) then *N* = 1 changed strategy - Parallel assembly (*N* = 6) - Use of the locking component (*N* = 3)	- Parallel assembly (*N* = 7) - Use of the locking component (*N* = 3)
Loading the pieces on the cobot table	- When one piece per category is on the cobot's table (*N* = 3) then cobot frequently stops - Move the piece after placed it on the table (*N* = 1). It causes error	- Move the piece after placed on the table (*N* = 1). It causes error
Other manifestations	- Manifestation of heat (*N* = 2)	- Hum (*N* = 1) - Rotation of some components of gearbox while waiting for cobot (*N* = 3) - Play with clips (*N* = 1)
Regard for the cobot	- React in advance (*N* = 1) - No awareness of cobot standing (*N* = 1) - Look to cobot while waiting (*N* = 8)	- No awareness of cobot standing (*N* = 1) - Look to cobot while waiting (*N* = 8)
Talk to operator	*N* = 2	*N* = 4

Regarding the **manifestation of tiredness**, some subjects show an increase in the number of manifestations during the same day (specifically, placing hands on hips, and sitting down); furthermore, most of the behaviors observed on the first day, emerge more frequently—in the same subjects—on the last day. In general, participants are often bored especially during the last day, which is characterized by longer waiting times. About **gestures with the hands**, over time from the first to the last day, a lower variability in the behaviors manifested and an increase in the frequency of manifestations have been noted. Furthermore, each participant is inclined to show a specific behavior (for example, touching the hair 2–3 times a minute). Considering the **assembly methods**, an adaptation to the task after the first moments of the first day can be noticed, whereby on the last day almost all the participants assemble the components in parallel. This leads to an increase in performance. Some observations are related to the preference of the participants in **loading the pieces on the cobot table**. The number of errors was reduced at the end of the week; the researcher's perception is that of an improvement in performance and a better awareness of the actions to be performed. For the “**other manifestations**” category, more variability and frequency emerged during the last day; the perception of the observer is that some participants implement behaviors to “fill the dead moments.” Concerning “**regard for the cobot**,” improvement in action planning during the week emerges and the participants tend to interrupt the actions they are carrying out to perform the joint action when the cobot is ready. In many cases, and during both the first and last days, the participants had to wait for the cobot. Interestingly, while waiting, participants almost always look at the cobot. Also, in some cases, they start looking in random directions after looking at the cobot for some time. We observed this gaze behavior directed toward the cobot in all participants of the NT group. Finally, the number of participants who **talk to the operator** in the room increased from the first to the last day.

**Figure 5:**
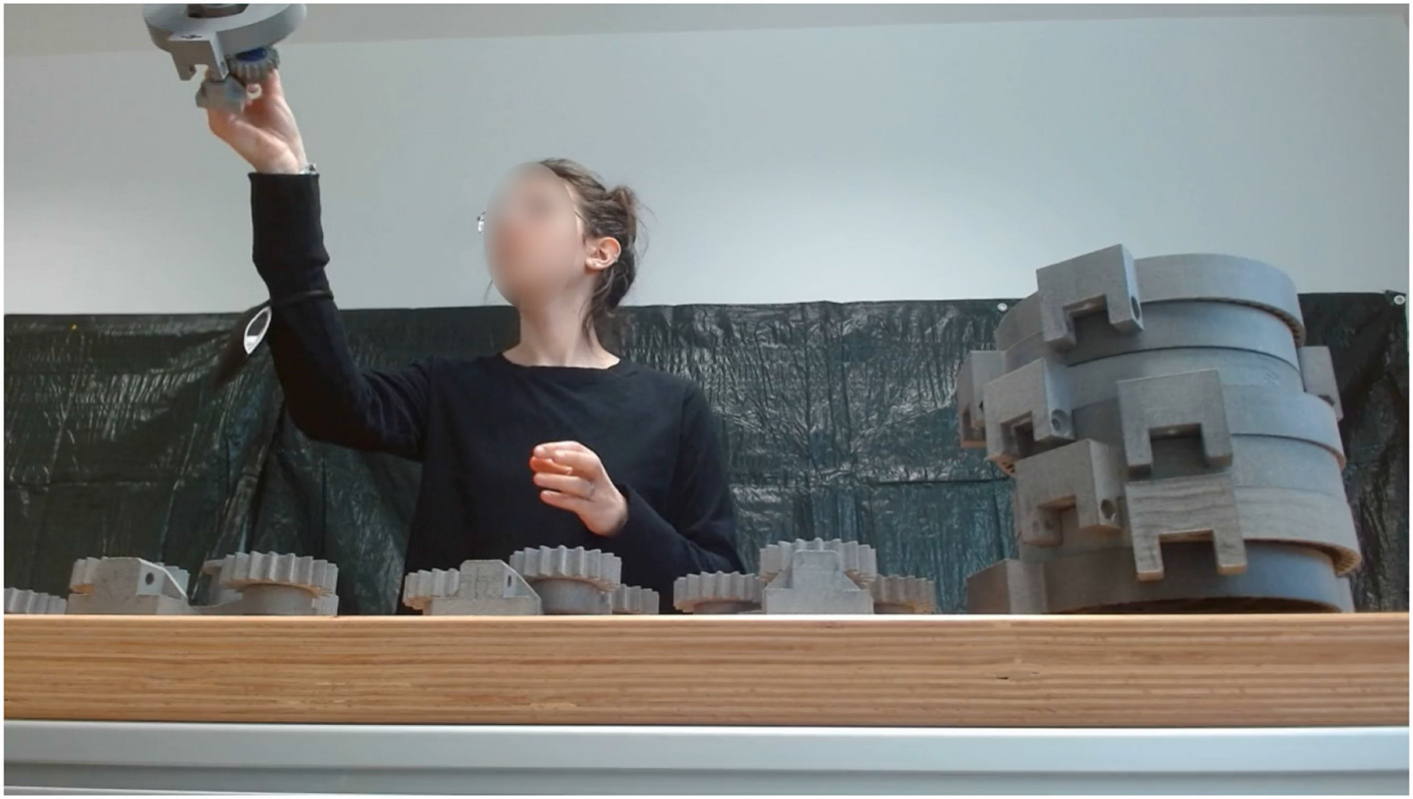
An illustration of a neurotypical participant collaborating with the cobot during the assembly task.

In conclusion, as observed, all participants assemble their parts faster than the cobot leading to a considerable amount of waiting time. After getting used to the task, the participants start gathering the multiple sub-assembly components (for future assembly) on the table, as well depicted in Figure 5 and, in almost all the instances, they preemptively assemble their parts. This process of adaptation to the task throughout the week can be noticed in all NT participants and leads to a generally increasing number of finished assemblies per day.

### 4.2. Participants with ASD

#### 4.2.1. Results from the observational grid

The videos collected from the eight ASD participants were also analyzed using the Observational Grid.

The results are summarized in Table 3 and are explained in more detail in the Supplementary material, paragraph 2. Some researchers' impressions which are difficult to understand by reading only the table are reported below.

**Table 3:** Summary of the observed behaviors related to the ASD group.

	**First day**	**Last day**
Manifestation of tiredness	- Lean hands or arms on table while waiting cobot (*N* = 4) - Arms crossed repeatedly - Hands on hips (*N* = 1) - Sit (*N* = 1) - Time monitoring (*N* = 4) - Stretch (*N* = 1) - Sigh (*N* = 1) - Yawn (*N* = 1)	- Lean hands or arms on table while waiting cobot (*N* = 3) - Arms crossed repeatedly (*N* = 2) - Sit (*N* = 1) - Hands on hips (*N* = 3) - Close eyes (*N* = 1) - Time monitoring (*N* = 5) - Stretch (*N* = 3) - Yawn (*N* = 1)
Gestures with the hands	- Rub fingertips (*N* = 1) - Rub face (*N* = 4) - Rub hands (*N* = 3) - Clap hands (*N* = 1) - Stereotypical hands' movements (*N* = 1) - Move the box to be emptied (*N* = 1) - Shake wrist (*N* = 1) - Touch glasses (*N* = 1)	- Rub fingers (*N* = 1) - Rub knuckles (*N* = 1) - Rub fingertips (*N* = 1) - Rub hands (*N* = 1) - Rub face (*N* = 4) - Touch glasses (*N* = 2)
Assembly methods	- Start assembling the gearbox as the participant take out the useful parts from the box (*N* = 2) - Empty the whole box before the assemblation (*N* = 6) - Sequential assembly (*N* = 4) - Parallel assembly (*N* = 3) - No assembly support used (*N* = 1) - Pieces are placed close together on the table (*N* = 1)	- Sequential assembly (*N* = 4) one changes strategy - Parallel assembly (*N* = 3/4) - No assembly supported used (*N* = 1)
Loading the pieces on the cobot table	- Add the piece anytime it is taken by the cobot (*N* = 1)	- Add the piece anytime it is taken by the cobot (*N* = 1)
Other manifestations	- Manifestation of effort (*N* = 2) - Greet the camera (*N* = 1) - Manifestation of heat (*N* = 2) - Wet the lips (*N* = 3)	- Manifestation of effort (*N* = 1) - Jump (*N* = 1) - Sway the body (*N* = 1) - Push components of the gearbox (*N* = 1)
Regard for the cobot	- Make the cobot wait (*N* = 5) - Look frequently at the cobot (*N* = 2) - Facial expression to react to cobot's action (*N* = 3) - Watch the cobot while it assembles the gearbox without preparing their part (*N* = 2) - React in advance (*N* = 1)	- Make the cobot wait (*N* = 3)
Talk to operator	*N* = 1	*N* = 1

**Figure 6:**
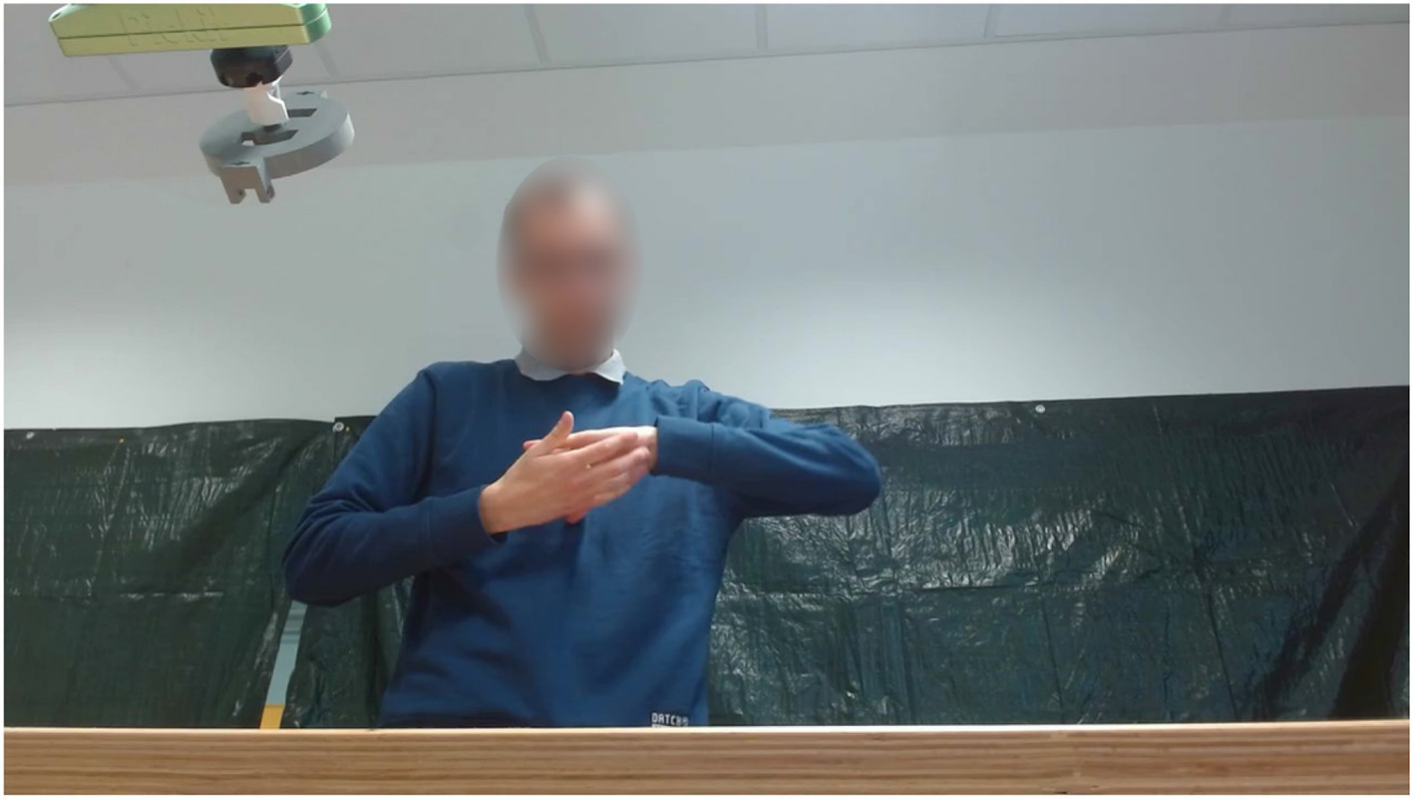
An illustration of a participant characterized by ASD performing some hand gesture while the cobot waits for the collaborative joint action.

As regards the manifestation of tiredness during the first day, it is possible to notice that the participants who rest their hands/arms on the table would have the possibility of “filling” the cobot's waiting time, for example by emptying a box. Furthermore, the behavior is usually gradual (one hand is placed, then two hands, then the whole arm is placed down). The researcher notes a general tendency to increase the same type of gesture in the same participant between the video recorded at the beginning and the end of the day, suggesting that these behaviors are related to fatigue. Conversely, the behaviors that are manifested on the third day, mostly similar to those that emerged on the first, do not increase in frequency during the day. Regarding **gestures with hands**, we note the emergence of some particular gestures; for example, a participant claps after a completed action, such as applause. Another moves his hands repeatedly as if it were a stereotypical gesture, or the hands are repeatedly scratched (see Figure 6), and in another case, a box is moved numerous times before finding the participant's preferred place. However, these gestures emerge only on the first day. Moving on to the **assembly method** category, we mainly observe two strategies: assembling one gearbox at a time, or in parallel. These strategies are the same ones that also emerge on the last day. A difficulty in changing one's strategy, even when not very effective, emerges but an increase in the speed of actions is observed. A frequent strategy related to loading pieces on the cobot table does not emerge, except the participant who immediately replaces the piece just taken from the cobot for assembly, reducing the risk of errors (Label 2, Figure 1). Furthermore, no differences emerge between the first and last day. Concerning **other manifestations**, the researcher notes in particular that gestures seem to appear in moments of boredom; further explanations can be found in the Supplementary material, paragraph 2. In the **regard for the cobot** category, it can be noted that some of the subjects fail to get good timing with the cobot, making it wait while performing other actions, or stopping to observe it while they could carry on with the work. Furthermore, visual expressions closely linked to the cobot's behavior emerge, such as amazement at its speed. There is generally an improvement in performance between the first and last day, with fewer empty moments. Finally, the number of participants who **talk to the operator** remain the same on the first and last day.

#### 4.2.2. Results from the unstructured notes

As mentioned, this paragraph contains annotations relating to the observed behaviors implemented by the participants with ASD.

Regarding the “**Task**” category, the most common challenges observed during the three work shifts were related to delays caused by: the lack of components loaded on the table; the need for technical intervention regarding issues s/he could handle independently; the cobot stops because of some mistake of the participant. On the other hand, remarkable behaviors ameliorating the task performance were: the participant is able to talk and work at the same time without being distracted; s/he is aware of the system functioning (e.g., knowing what to do when the cobot cannot detect a component or being able to use the pedals properly) and autonomous in task management (e.g., s/he knows how to rearrange the workstation after the cobot is being restarted).

Regarding the “**Work organization**,” participants were able/not able to: refill the table while the cobot is performing its cycle; to have his/her sub-assembly ready when the cobot approaches to collaborate; to organize multiple sub-assemblies to get ahead of the assembly work and to take advantage of cobot stops to arrange components on the desk. The last three aspects within the “Work organization” comprise break management, the end of the shift management, and physical fatigue. The break is, in some cases, taken autonomously by the participant, while, in other cases, the researchers have to remind the participants (totally immersed in the task). As for the end of the shift, the idiosyncrasy against incompleteness leads the participant to finish the box already started (and containing five pieces each) or to finish the pieces on the desk (leaving the table empty). To reduce physical fatigue, some participants used a chair to sit down for a while.

Coming to the “**Say—quotes**” category of behavior observed in ASD participants, their verbalizations were grouped for similarity of concepts (below are reported only the ones conveying aspects not already mentioned in the other categories). Table 4 can be used to go into details of the quotes grouped by “anthropomorphism,” “attention to details,” “control/feedback,” and “general opinion on the task.”

**Table 4:** Quotes collected from the participants during the week.

**Say quotes**	**Citation**
Anthropomorphism	- “Does the robot have a name?” “Its name is given from the factory, it is Fanuc”
	- “Come on FANUC come on!” (referring to the cobot one more time looking for the parts it cannot find)
	- “I am sorry that you are waiting” (referring to the cobot) “How empathetic you are” (He smiles back)
	- “Very good, go robot”
	- “Come on, there are three beautiful little pieces... Now I'm going to move it for you sweetie”
Attention to details	- “This piece is defective” (he realizes that one piece is slightly different from the others)
	- “I have discovered something: The best placement of components is on the left side of the buffer”
	- “Maybe that's why he's having a hard time catching it” (the operator notices that one component is darker in color)
	- “I realized that by putting the smaller rings near the edge the cobot was not taking them”
	- “Is it not slower than yesterday?” (The operator reports that the cobot is slower in opening the pincers)
Control/feedback	- “I need to calculate how long it takes me to do an assembly so that I will not leave any pieces for my colleague”
	- “I made half of this box, at the end of the week can you tell me how many pieces I made on average?”;
	- “What box did they take away? Which were the first boxes that you brought to me?”
	- “Will you count the assemblies or shall I count them?”
General opinion on the task	- “While doing this work, those who are not Aspergers become so”
	- “I was told that you were the one that collaborated with me”
	- “So, I assemble and you disassemble”
	- “It is relaxing for me to do this stuff, I don't think while I am working, I have less pressure”

About “**Act—Recurrent behaviors**,” here a list of the most interesting notes is reported: looking at cell phone; putting on headphones with music; leaning on the table; stretching; puffing; yawning; sitting; giggling; humming; keeping time with the foot; chatting (also talking to self); moving hands (flickering) and snapping fingers.

Finally, the “**Feel—Emotional expressions (if any)**” category summarizes the following manifestations. First of all, nervousness is generated by: the participant's fatigue in joining the two sub-assemblies; cobot stops that last for a long time (forcing the operator to prolonged inactivity); work interruption caused by phone notifications; the cobot that fails in detecting a component for several times consecutively; failure to finish the work shift by completing the box already started or finishing all the pieces on the table (leaving the table empty). Additionally, boredom/tiredness manifests in puffing, slumping on the table, yawning, or sighing. Lastly, other notable manifestations were: happiness (s/he smiles, listens to music amused, dances, giggles, hums), a sense of safety (s/he is not afraid of proximity to the cobot), and fear (s/he jumps when the cobot approaches him).

### 4.3. Comparison

Some differences between the two groups emerge from the observations made both from a qualitative and quantitative point of view.

#### 4.3.1. Qualitative comparison

In general, a greater number of manifestations of tiredness and hand movements are noted in participants with ASD. In particular, it is noted that behaviors related to fatigue also emerge in the NT group, but later than in the ASD group. Participants characterized by ASD show some signs of boredom in the very first moments of the interaction; in particular, there are many instances in which the user looks at his/her watch while the robot is performing its activities.

Considering hand gestures, more stereotyped movements and rubbing of the fingertips or hands emerge in the ASD group, while the NT group tends to move their hands over their body: face, hair, and glasses.

Even though the assembly methods adopted are similar in the two groups, it is observed that NT participants have a faster adaptation to the task, especially in terms of sequence, timing, and positioning. Nevertheless, it should be noted that even in the group characterized by high-functioning autism some participants also showed an improvement in performance and therefore a change in the assembly methodology.

Considering the “other manifestations” category, a wider variety of behavioral productions can be noted in the NT group compared to participants with ASD. Moreover, in the ASD group, these actions are linked to specific moments (for example, a difficulty), while for the NT participants, they are more pervasive. Furthermore, it appears that participants with ASD engage in behaviors in which their body is the protagonist (e.g., greeting, frowning, jumping); on the other hand, in the NT group, the actions usually involve an external tool (a clip, one of the components of the gearbox, etc.). In both cases, self-facing gestures increase over time, and presumably with increasing fatigue. It can also be observed that NT participants tend to talk more with people in the room than participants with ASD.

Regarding the attitudes toward the cobot, it is noted that the group of ASD participants is less inclined to adapt: there are more situations in which the cobot is ready to collaborate but the participant has not completed the sub-assembly. This behavior could be explained by the difficulty of users with ASD to work in parallel on different assemblies (multitasking), well-known in the literature (Mackinlay et al., 2006; Yang et al., 2017). In general, it can be noticed that the adaptation process observed for NT participants emerges less in the participants with ASD, as they maintain their work routine almost identically throughout the week. As a result, the total number of assembled components is lower and increases less throughout the week, as later confirmed by the quantitative comparison reported in Section 4.3.2. These aspects are a direct consequence of the robot's waiting time. The participant with ASD usually did not show any urgency in responding to the robot when it brought its sub-assembly. As mentioned before, in many instances, they finish the assembly after the robot arrives for the final joining of sub-assembly parts. In the NT group, on the other hand, there is a decrease in the moments of pause, with a consequent increase in performance. We can therefore deduce that ASD participants don't prioritize the robot or the final joining task and continue doing what they are doing, even if it is gathering the components for future assembly. This is in contrast with our observations related to NT participants, who prioritized the robot over other sub-tasks, which led to negligible waiting time for the robot. However, this observation should not be considered in an absolute sense. Some participants characterized by high-functioning autism demonstrated flexibility and were able to both carry on with the work and be ready when the cobot approached and showed multitasking skills. What changes, once again, is the number of participants who show this behavior. Within the group with ASD, there is greater variability in the manifestations observed, so it was possible to identify high-performing participants and less flexible and low-performing participants. The NT group, on the other hand, was more homogeneous in the observed behaviors. This observation indicates that there could be differences in the best synchronization logic between the user and the robot when dealing with NT or ASD workers.

Interestingly, the ASD group showed more variability in their facial expressions than the NT group in response to the cobot actions. Moreover, interesting points of discussion come from the analysis of gaze patterns. Considering the ASD group, gaze, even if directed toward the cobot, did not result in the adaptation of their actions. Furthermore, the participants with ASD did not have a clear pattern of looking at the robot while waiting. On the other hand, the NT group looked at the cobot more often during the task either because waiting for it or to better time their assembly schedule.

It has also been noted that sometimes participants with ASD prefer to maintain distance from the robot throughout the sessions. This is particularly evident in the timing of loading components onto the cobot table. The NT participants gather robot components whenever they deem necessary and don't mind working closely with the robot. The participants with ASD, instead, tend to gather the components for the robot after the robot brought its part for final joining, i.e., when the robot is not working on its side of the table (see Figure 1), further adding to the waiting time of the robot. This space factor needs to be taken into account when allocating collaborative sub-tasks to participants with ASD to yield better performance.

Finally, some aspects are shared among the two groups. First of all, it emerges that there is not one behavior more frequent than another by looking at the participants as a whole, but a personal tendency to implement the same behavior repeatedly, whatever it may be (e.g., moving hands in a certain way, touch a certain point of the face, touch the components of the gearbox, look at the time). Furthermore, in both groups, the moments of boredom and waiting are characterized by a greater number of hand gestures and are often associated with bored expressions or yawns.

#### 4.3.2. Quantitative comparison

First, to assess and compare the duration of different actions, we analyzed the data obtained from the annotated labels. Interestingly, results show considerable differences between the two groups in this sense.

**Figure 7:**
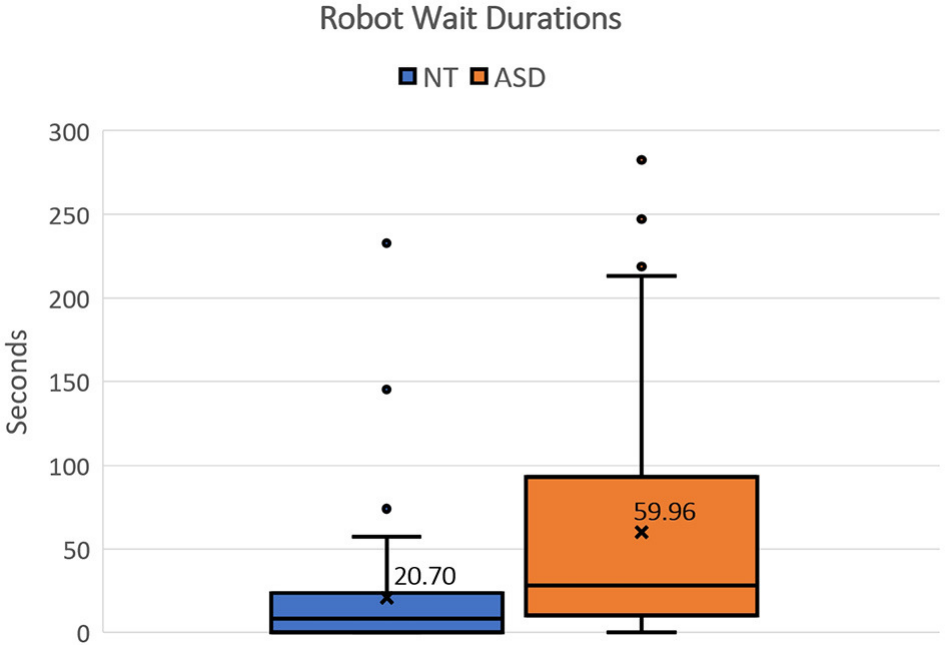
Box plot showing the distribution of Robot Wait duration for NT and ASD groups.

One of the quantitative measures that differed significantly is the robot's waiting time. As already observed in the qualitative comparison in Section 4.3.1, most of the participants with ASD displayed a lower sense of urgency in attending to the robot. To quantify this, we calculated the average waiting time of the robot across all sessions for NT participants and participants with ASD. The average waiting time for NT participants was found to be 20.7 s per video, while for the participants with ASD, the wait duration was almost triple at 59.96 s per video. Figure 7 visualizes the box-plot of the robot's waiting time from each annotated video for NT and ASD groups. To determine if this difference is statistically significant, we visualized the mean robot's waiting time for each participant using Q–Q plots. We found that the data does not follow a normal distribution and thus violates the normality assumption of the independent samples *t*-test. Hence, we chose to run the Mann-Whitney test and found a significant difference (*U* = 11.0, *p* = 0.016).

**Figure 8:**
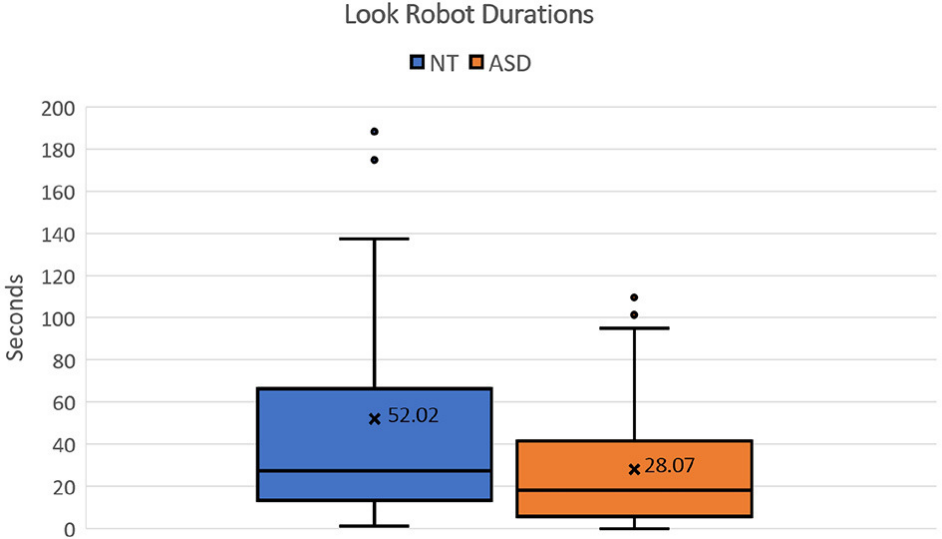
Box plot showing the distribution of Look Robot duration for NT and ASD groups.

Some differences were also qualitatively highlighted in terms of gaze patterns. In these terms, first, we confirm that considerable differences exist in the amount of time participants spent looking at the robot, as shown in Figure 8. On average, NT participants spent 52.02 s per video looking at the robot, whereas the participants with ASD spent only 28.07 s per video. NT participants spent almost double the amount of time looking at the robot compared to the participants with ASD. This indicates a disparity in visual engagement with the robot between the two groups. Secondly, during the annotation process, we noticed additional differences related to the duration of continuous gaze contact with the robot for the participant with ASD. NT participants tended to have longer periods of continuous gaze contact. In contrast, the participants with ASD had shorter periods of gaze contact and frequently looked away. To measure this disparity, we calculated the maximum duration of gaze contact with the robot throughout the sessions. For the participants with ASD, the average value of the maximum gaze contact period was 7.93 s per video, whereas the mean value for NT participants was 12.49 s per video. These observations regarding gaze duration align with previous research by Damm et al. (2013), where they found a significant decrease in gaze contact with social robots among individuals with ASD over the course of a session. Similar to the robot's waiting time, we visualized the mean values for each participant as Q–Q plots. Neither the look-at-robot duration nor the maximum gaze contact period followed a normal distribution. The Mann-Whitney test yielded a significant difference in look-at-robot duration (*U* = 15.0, *p* = 0.042). However, the maximum gaze contact period did not result in a significant outcome (*U* = 19.0, *p* = 0.095). This indicates that a larger sample size might be required to effectively detect smaller effects.

Focusing now on the performance analysis computed over the full experimental week, some additional differences seem to arise between the two groups.

**Figure 9:**
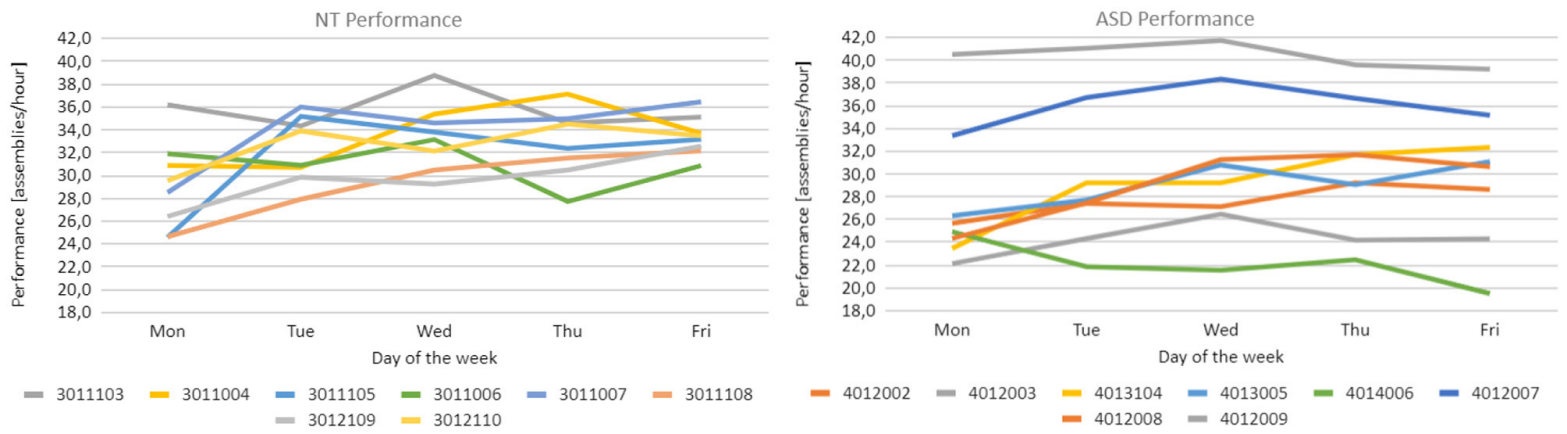
**(Left)** Each line represents the performance index of a member of the NT group over the experimental week, **(Right)** the same is represented for the members of the ASD group.

The results collected for the NT group, depicted on the left side of Figure 9, clearly follow a trend of increasing performance for all the members of the group and the tendency to converge to a common top performance. In fact, in terms of the average performance index, the results show a relevant increase over the week (+15%), going from 29.08 assemblies/hour on Monday to 33.43 assemblies/hour on Friday. Moreover, looking at the daily standard deviations computed using the daily performance indexes of each member, results decrease from 3.95 to 1.73 suggesting that the participants tend to converge toward a common level of top performance by the end of the experimental week.

The results collected for the group characterized by ASD, depicted on the right side of Figure 9, again follow a moderately increasing trend over the experimental week (+9%), going from 27.59 to 30.11 assemblies/hour (Monday to Friday). However, it can be noticed that the performance trends of each member of the group are quite spread apart in the plot and do not show any tendency to converge or diverge during the experimental campaign. Looking at the daily standard deviations computed over the daily performance indexes of all the members of the ASD group, results remain pretty stable, oscillating between a minimum of 5.75 and a maximum of 6.52.

**Figure 10:**
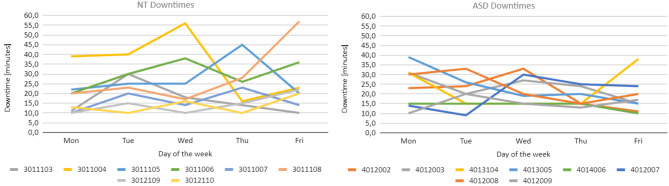
**(Left)** Each line represents the minutes of downtime of a member of the NT group over the experimental week, **(Right)** the same is represented for the members of the ASD group.

To further analyze the actual performance of each participant, Figure 10 reports the trend of daily downtime for each participant: on the left, the data related to the NT group is presented while, on the right, the data of the ASD group can be found. As already mentioned in Section 3.5.4, downtimes are made up of both breaks requested directly by participants and unexpected stops that required a restart of the system. On this basis, one may think that the actual duration of daily downtime could affect the level of tiredness of the participant and consequently the achieved performance level. However, looking at the individual trends of both performance and downtime, this hypothesis is not confirmed. For brevity, only the data collected for participant number 3011044 is discussed here, since it is the one with the most variable trend of downtime, but the same conclusion can be drawn also for the other participants. Considering Figures 9, 10, participant 3011044 experienced a relevant increase in downtime between Tuesday and Wednesday and achieved an increased performance level. However, the same participant also experienced a huge drop in downtime between Wednesday and Thursday but, once again, an increased performance level was achieved. Considering this, we can conclude that the duration of downtime does not seem to affect the trend of performance.

**Figure 11:**
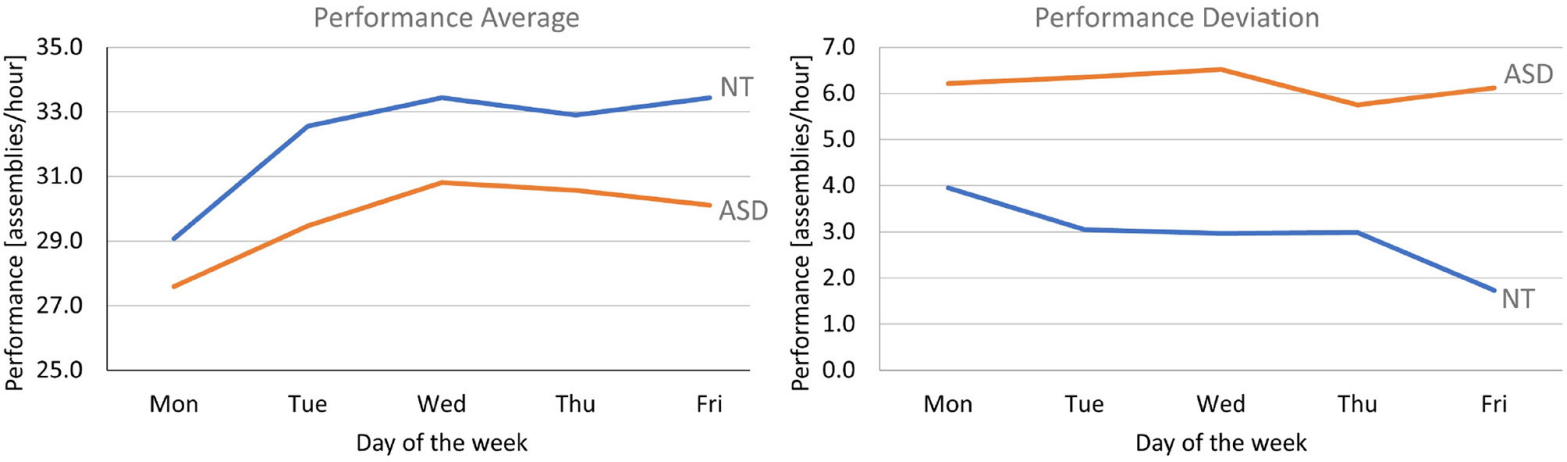
**(Left)** The comparison between the average performance of the NT and ASD groups. **(Right)** The comparison between the standard deviation of the same groups.

To perform a direct comparison between the two groups, the assumption of normal distribution first has to be verified. To do so, we first looked at the Q–Q plots and then performed a Shapiro-Wilk test (NT—*p* = 0.490, ASD—*p* = 0.094). Since the data for both experimental groups is confirmed to be normally distributed, an ANCOVA test was performed to analyze the time*group influence effect over the collected performance indexes. Results confirm a statistically significant difference between the two groups (*F* = 4.85, *p* = 0.010). In more general terms, the collected data clearly shows that the rate of improvement achieved by the NT group (+15%) is considerably higher than the one achieved by the ASD group (+9%), as depicted on the left side of Figure 11. On average, the absolute number of assemblies/hour reached by the NT group remains higher than the ASD group for every day of the experimental week. However, it is interesting to notice that both the best and the worst performers among all the participants belong to the ASD group. The range of minimum and maximum performance for the NT group stands between 24.57 and 38.75 assemblies/hour, while for the ASD group, the same range spans between 19.50 and 41.74 assemblies/hour. This is consistent with what was reported in the qualitative analyses, namely that in the ASD group there is greater variability in behaviors, while the NT group is more homogeneous. Finally, the tendency of the NT group to converge to a common best performance level is interestingly not reflected in a similar trend for the ASD group, as shown in the right side of Figure 11.

## 5. Conclusion and future works

Our goal was to explore the different needs of NT participants and participants with ASD during collaboration with a cobot. To this end, we collected video recordings of both experimental groups working in a robotic collaborative assembly cell reproduced in a lab environment. We used the NOVA tool, to annotate the videos and analyze them quantitatively. Moreover, both an *ad-hoc* observational grid and unstructured note-taking were leveraged to collect qualitative points of discussion. It must be said that no measurement of the degree of agreement between the different observers was carried out even if the results collected were mutually consistent. We found some key differences between NT and ASD participants in terms of prioritizing the robot partner, gaze patterns, facial expressions, multi-tasking, and personal space. While our findings are generally in line with existing literature on ASD participants in social settings, it was surprising that it applies to a context that is not so obviously social (no other humans or humanoid robots/agents).

The participants with ASD exhibited a lower sense of urgency in responding to the robot. They tend to complete the ongoing sub-task before attending to the robot. This behavior led to long waiting periods for the robot. This result is consistent with what is reported in the literature, namely that subjects with ASD have difficulties in prioritizing tasks (Murin et al., 2016). On the contrary, NT participants prioritized the robot and the final joining activity, which led to negligible waiting time for the robot. This difference in prioritizing the robot plausibly affected the assembly performance as the ASD group completed fewer assemblies on average compared to the NT group, as confirmed by the quantitative comparison provided.

Regarding gaze patterns, both groups of participants tend to gaze toward the robot, although the duration of gaze contact is different. As noted by Zhang et al. (2017), gaze information can improve synchrony and communication in human-human collaboration. In our case, the gaze behavior can be considered as a cue to let the collaboration partner (in this case, the robot) know that they have completed their part of the task, which could be useful to adapt the behavior of the robot and to improve the collaboration experience of the user.

Interestingly, the ASD group reacted more frequently than the NT group with facial expressions to the cobot actions. This result could confirm a particular interest of people on the spectrum toward robotic technology even in industrial settings and opens up interesting research questions related to the exploitation of facial expressions in similar scenarios. To date, it's well-known that children with ASD, the segment of the population on which most studies of this type are concentrated, have great interest in robots (Alves et al., 2020). First of all, this preference is related to the fact that robots, unlike people, operate within predictable systems and provide a highly structured environment that allows individuals with ASD to be more focused and feel comfortable (Takata et al., 2023). Secondly, as also underlined in Atherton and Cross (2018), individuals with ASD show a tendency toward anthropomorphism and greater empathic skills when interacting with non-humans, namely robots. Individuals with ASD are more at risk for feelings of loneliness, and feel themselves lacking in their social skills (Jobe and White, 2007); interaction with a robot has less emotional risk, and this could explain the greater tendency of participants with ASD to react and anthropomorphize the robot of out scenario.

In terms of assembly performance, both groups generally improved over the week even though at a higher rate for the NT participants. This seems to suggest that a learning curve was experienced by both groups during the first days of the week while, during the last days, only the NT group optimized their working pattern (e.g., multitasking) to reach even better performance levels. This is also confirmed by the tendency of the NT group to converge to a common maximum performance index representing the saturation related to how the task was set up. On the contrary, each member of the ASD group kept pretty much the same working pattern, therefore, limiting their performance level to the “goodness” of their strategy. Nevertheless, as already mentioned, we observed in the group with ASD a greater variability and a potential in the expression of multitasking skills and flexibility. This fact suggests that participants with ASD potentially have the skills to perform well. It would be appropriate to propose specific training or to accustom the participants to the task, to support this potential (i.e., multitasking) which, by itself, emerges with more difficulty.

In terms of personal space, we noticed that participants with ASD preferred to maintain a distance from the robot throughout the sessions while the NT group generally did not mind working closely with the cobot.

The outcomes of this study hold profound implications for both Industry 4.0 and the broader societal context. The observed performance of a specific individual with ASD surpassing their neurotypical counterparts, despite the overall lower performance of the ASD group, underscores the immense potential for inclusivity within Industry 4.0 environments. Furthermore, the intricate balance of similarities and high variations within the ASD group reaffirms the spectrum nature of autism. As we move forward, embracing a personalized approach that caters to individual traits and preferences becomes paramount, particularly in designing adaptive robot behaviors and task allocations. Moreover, the significant behavioral differences identified between the neurotypical and ASD groups emphasize that solutions designed for the former may not align effectively with the needs of the latter.

In this study, we adopted an exploratory approach to identify behavioral patterns during a collaborative assembly task. As such, we did not specifically elicit responses to certain scenarios or investigate how participants from the NT and ASD groups would differ in their reactions to specific events, such as mistakes made by the robot or handling stressful situations. However, it is important to recognize that such situations can significantly impact participants' responses and behaviors. Exploring these aspects in future studies could provide valuable insights related to how individuals with different needs might react and cope with such scenarios.

As mentioned, this study revealed higher variability in the observed manifestations and performance in the ASD group than in the neurotypical group. In future works, it would be interesting to understand whether this variability is related to any particular personal traits. To this end, objective and self-reported data relating to personal characteristics could be collected.

Furthermore, conducting focused studies that incorporate multimodal data has the potential to provide a more comprehensive understanding of participants' behaviors and interactions. Finally, it would also be of great interest to undertake additional experimental campaigns with more participants and in actual company-based settings in order to validate the presented results even with data collected “in-the-wild.”

## Data availability statement

The datasets for this article are not publicly available due to concerns regarding participant/patient anonymity. Requests to access the datasets should be directed to the corresponding author.

## Ethics statement

The studies involving humans were approved by Ethics Committee of Istituto di Ricovero e Cura a Carattere Scientifico Eugenio Medea. The studies were conducted in accordance with the local legislation and institutional requirements. The participants provided their written informed consent to participate in this study.

## Author contributions

Study conception and design: MMo, PP, and ML. Data collection: ML, MC, and EM. Analysis, interpretation of results, and draft manuscript preparation: MMo, PP, ML, and MC. Supervision and proof-reading: FS, RV, EA, and MMa. All authors reviewed the results and approved the final version of the manuscript.
