# Supplementary material for: Behavioral patterns in robotic collaborative assembly: comparing neurotypical and Autism Spectrum Disorder participants
Source: Front Psychol. 2023 Oct 26;14:1245857. doi: 10.3389/fpsyg.2023.1245857 (PMC10637657; doi:10.3389/fpsyg.2023.1245857)
Supplement: Supplementary file 1 [file Data_Sheet_1.PDF]

Supplementary Material

Here we provide a detailed description of the observed behaviors of NT and ASD groups using the Observational Grid tool.

1 RESULTS FROM THE OBSERVATIONAL GRID FOR NT GROUP

Regarding ”**manifestation of tiredness**” on the first day, the participants tend to lean their hands or arms on the table while waiting for the cobot (N=5). Sometimes (N=3), this behavior is accompanied by hand activities that appear to be used to “fill in” time, namely turning a gearbox piece, playing with the hands, and tapping the fingers on the table. N=2 participants place their hands on their hips. Only in one case, the behavior is gradual and the participant leans more frequently over time. One participant sits while waiting, increasing the frequency of the behavior throughout consecutive recordings. About time monitoring, N=2 participants repeatedly look at the clock, one of which also uses a mobile phone to look at the time. N=2 participants stretch their necks and chest. A tendency to increase these behaviors in the same participant between the video taken at the beginning of the day and the last one is noted. On the last day of the video, an increase in behaviors related to tiredness and boredom is perceived, and these manifestations correlate with increasing empty moments in which there are no more parts of the gearbox to assemble. Again, most fatigue behaviors were related to placing hands on the table or the body (N=8). Only one participant sits down, albeit frequently. N=4 participants monitor the time with the mobile phone or the watch. One participant performs neck stretches. N=2 participants yawned during the experience, numerous times, and one participant snorted. Therefore, an increase in these manifestations was found in NT participants, who did not show fatigue in such a direct way during the first day. In general, it’s noticed that the participants are often bored, plausibly because of the considerable waiting time.

Regarding ”**gestures with the hands**”, participants show rubbing of the fingertips (N=1), the face (N=4), and the hands (N=2); N=3 frequently touching their hair. Behaviors implemented by a single participant are those of pulling up the sleeves of the sweatshirt, touching the glasses, the wrist (the watch), and adjusting the clothes. On the last day, we note that some attitudes are reported by a greater number of participants, but are less varied: touching hair (N=3), face (N=6), and glasses (N=3). One participant (N=1) taps the watch. In general, an increase in these gestures in all participants during the experience is noted; each participant is inclined to show a specific behavior (e.g., touching the hair 2-3 times a minute).

Considering the ”**assembly methods**”, N=3 participants start assembling the gearboxes as they take out the useful parts from the box; One participant starts by first emptying the whole box and then proceeds with the assembly, but s/he changes strategy during the day. N=3 participants built the gearbox sequentially, waiting before completing the collaboration with the cobot, but one of them changed the method during the day; therefore, at the end of the day, N=6 participants assembled the pieces in parallel. Of these, N=3 participants already use the locking component in the construction of the piece, anticipating this action usually carried out after the joint with the cobot. One participant also shows particular multitasking skills: while s/he is waiting for the cobot with the gearbox in the hand, with the other hand s/he mounts a new gearbox. On the last day of the study, nearly all (N=7) participants adopted a parallel assembly strategy. In general, an adaptation to the task is noted with a consequent increase in performance.

Some observations are related to the preference of the participants in ”**loading the pieces on the cobot table**”. N=3 participants prefer to fill the table when the cobot has only one piece available per category on

its table. In one case, the participant moves the pieces after placing them back on the table, causing the cobot to fail and stop. At the end of the week, the number of errors is reduced; only one participant moves the pieces on the table several times after having placed them. Also in this case, the perception of the observer is that of an improvement in performance and a better awareness of the actions to be performed.

In the ”**other manifestations**” category, the only behavior that emerges on the first day is in N=2 participants who manifest heat, waving their hand or shirt. On the last day, however, some participants implement behaviors to “fill the dead moments”: one participant hums, N=3 participants rotate some components of the gearbox while waiting for the cobot, and one plays with the clip needed to close the gearbox.

Concerning ”**regard for the cobot**”, one participant (N=1) constantly reacts in advance to the action of the cobot, holding the piece in hand before its arrival; one participant does not look at the robot during the task and does not notice that it is waiting for the joint. On the last day, again one participant is not aware of the cobot standing, while another participant (N=1) has no pieces ready for the joint when the cobot arrives. In one case, the participant prefers to empty the box than perform the joint, while talking to someone in the room. We observed a gaze behavior directed toward the cobot in the whole group (N=8).

In total, N=2 participants often ”**talk to someone**” in the room during the first day, and the number increased to 4 on the last day.

2 RESULTS FROM THE OBSERVATIONAL GRID FOR ASD GROUP

Regarding ”**manifestation of tiredness**” during the first day, a behavior manifested by N=4 participants is that of placing their hands and/or arms on the table in moments of waiting. We note that this behavior is present when the waiting time could be filled by working, for example by emptying a box. In one case, the participant crosses his/her arms repeatedly or rests them on his/her hips. Usually, this behavior is gradual, i.e. the participant starts by placing one hand, then two hands, and then placing the arms. One participant sits while waiting. Another frequent behavior is to look at the time using the clock (N=3) or the mobile phone (N=1) while waiting for the cobot; in the case of the participant looking at the phone, the action is repeated 8 times during the 3 videos. Less frequent behaviors are related to stretching (N=1 participant but repeated 3 times), sighing (N=1), and yawning (N=1). There is a general trend to increase these gestures in the same participant between the video taken at the beginning and the end of the day, suggesting that these behaviors are related to tiredness. The manifestations related to fatigue are similar during the last day. In particular, N=3 participants place fists/hands on the table; in N=4 cases the hands are resting on a part of the body (arms crossed, on the hips, etc.); we note that a participant, during this kind of rest, closes his/her eyes for a few seconds. N=5 participants look at their watch/mobile phone several times during the same video. N=1 participant sits waiting, while another participant (N=1) yawns. N=3 participants perform stretching. In general, no gradual increase in behaviors associated with fatigue throughout the day emerges, compared to the first day.

As far as ”**gestures with the hands**”, N=3 participants rub their hands more than once during the videos recorded on the first day, of which one constantly rubs fingertips and one claps the hands after rubbing them; we note that this last action takes place after having completed a gearbox, as a sort of celebration. N=4 participants rub their faces numerous times during the videos. One participant performs frequent and stereotypical non-purposeful hand movements. In one case, the participant shakes the wrist with the watch frequently, as if it bothers him/her. One participant moved the box to be emptied three times before placing it firmly on the table. Another participant (N=1) touches the glasses throughout the experience. Regarding

the last day of the experiment, frequent gestures are the rubbing of the fingers (N=1), the knuckles (N=1), the fingertips (N=1), the hands (N=1), and the face (N=4). Also, N=2 participants touch their glasses frequently. In general, it is noted that each participant implements characteristic and preferred behaviors and that the most frequent one is touching parts of the face with the fingers.

Moving on to the "**assembly method**" category, a few strategies can be observed. Some participants (N=6) first empty the box with the pieces completely, and then move on to assembling. On the contrary, N=2 participants start assembling the gearbox as they take out the useful parts from the box. However, in some cases, this leads to having many gearbox halves started but not yet completed. Other strategies implemented by the participants are either to build one piece at a time (N=4), or in parallel (N=3). In one case (N=1), the participant does not use the support for assembly, and this allows him/her to be faster. We also note that one participant places all the pieces extremely close together, for faster assembly. On the last day of the experiment, the assembly methods remained the same as on the first day, except one participant who started by building the pieces sequentially and then moved on to a more efficient parallel assembly strategy. In general, an adaptation to the task, with regards to the speed in performing actions, is noted, but a difficulty in passing from a less efficient to a more efficient assembling method emerges.

Participants in this group showed no preference for strategies related to "**loading pieces on the cobot table**". The only exception is that of one participant (N=1), who always adds a piece as soon as it is taken by the cobot. Thanks to this approach, in this case, there were always at least two available pieces of that type on the table of the cobot leading to fewer unexpected stops. No differences were noted in this sense between the first and last day of the experimental week.

Regarding "**other manifestations**", N=2 participants show effort during the exercise, frowning and pursing their lips, during some assembly difficulties. One participant greets the camera during a moment of stasis while another participant (N=1) shows feeling hot. We have included the action of wetting the lips with the tongue in this category: In one case, this action is performed frequently and incrementally from the first to the last video. On the last day, only one participant (N=1) shows effort during some moments, while another (N=1) jumps in place. One participant sways with the body during the task, and another one (N=1) pushes the components around while the cobot is waiting. The perception of the researchers is that these gestures are performed out of boredom, to fill moments in which there is nothing to do.

In the "**regard for the cobot**" category, an attitude that frequently emerges is that of making the cobot consciously wait when it arrives for the joint action (N=5), by finishing emptying the box or preparing new gearboxes even though there are already pieces ready for the final assembly. N=2 participants frequently look at the cobot, both during assembly and during breaks; the researcher thinks that the looks are given for some sort of monitoring of when the cobot should arrive. N=3 participants show facial reactions to the cobot actions, and in particular astonishment (N=1), and disappointment (N=2) for the cobot errors or speed. N=2 participants do not adapt their mode of action to the timing of the cobot: in fact, the participants watch the cobot while it mounts its half, but when it arrives for the joint action, they do not have any pieces ready. One participant (N=1), on the other hand, shows marked improvement in this aspect, learning to anticipate the cobot, and picking up the piece to be combined a few seconds before the cobot arrives. On the last day of observation, some participants tended to make the cobot wait less (N=3).

In total, one participant frequently "**talks to someone**" in the room on the first day, and the number remained the same (but a different person) on the last day.
